# Advances of the reverse lactate threshold test: Non-invasive proposal based on heart rate and effect of previous cycling experience

**DOI:** 10.1371/journal.pone.0194313

**Published:** 2018-03-13

**Authors:** Leonardo Henrique Dalcheco Messias, Emanuel Elias Camolese Polisel, Fúlvia Barros Manchado-Gobatto

**Affiliations:** School of Applied Sciences, University of Campinas, Limeira, Sao Paulo, Brazil; Universitat de les Illes Balears, SPAIN

## Abstract

Our first aim was to compare the anaerobic threshold (AnT) determined by the incremental protocol with the reverse lactate threshold test (RLT), investigating the previous cycling experience effect. Secondarily, an alternative RLT application based on heart rate was proposed. Two groups (12 per group-according to cycling experience) were evaluated on cycle ergometer. The incremental protocol started at 25 W with increments of 25 W at each 3 minutes, and the AnT was calculated by bissegmentation, onset of blood lactate concentration and maximal deviation methods. The RLT was applied in two phases: a) lactate priming segment; and b) reverse segment; the AnT (AnT_RLT_) was calculated based on a second order polynomial function. The AnT from the RLT was calculated based on the heart rate (AnT_RLT-HR_) by the second order polynomial function. In regard of the Study 1, most of statistical procedures converged for similarity between the AnT determined from the bissegmentation method and AnT_RLT_. For 83% of non-experienced and 75% of experienced subjects the bias was 4% and 2%, respectively. In Study 2, no difference was found between the AnT_RLT_ and AnT_RLT-HR_. For 83% of non-experienced and 91% of experienced subjects, the bias between AnT_RLT_ and AnT_RLT-HR_ was similar (i.e. 6%). In summary, the AnT determined by the incremental protocol and RLT are consistent. The AnT can be determined during the RLT via heart rate, improving its applicability. However, future studies are required to improve the agreement between variables.

## Introduction

Over the last fifty years, the anaerobic threshold concept (AnT) has been used inside the sports sciences and clinical contexts. This concept was initially proposed for cardiac patients [1], but its application is also considered for control and prescription of individualized exercise intensity in trained individuals [2]. Physiological parameters such as respiratory and metabolic are commonly used to identify the AnT [3]. In this sense, the Maximal Lactate Steady State protocol (MLSS) [4, 5] is considered the gold standard protocol for this determination. The MLSS has been characterized as the highest intensity that may be supported over time without blood lactate accumulation [5]. However, the necessity of several evaluation days hinders the MLSS application.

Conversely, single-session tests such as the incremental protocol (i.e. graded exercise test) are valid alternatives for the AnT identification. During this application, the elevation of the intensity increases the pyruvate rate production that eventually exceeds the maximal rate of pyruvate oxidation, resulting in the formation of lactate. Thus, the AnT is identified during an incremental protocol based on the non-linear increase or a distinct change in the inclination of blood lactate curve [6, 7]. Although mathematical procedures like bissegmentation of two linear regressions [8, 9], fixed blood lactate concentration [10] or maximal perpendicular distance [11] were proposed and extensively used to overcome disadvantages of the visual identification [12], criticism still persists regarding the AnT determination during the incremental protocol [13, 14].

Based on such criticisms, Dotan [15] proposed a novel-testing coined as Reverse Lactate Threshold test (RLT). This application is conducted in two complementary phases. During the lactate priming segment phase (phase 1) individuals are submitted to a controlled warm-up (i.e. 2–3 stages) followed by two subsequent stages at and above the predicted AnT intensity, respectively. Thereafter, during the reverse segment phase (phase 2) the intensity is stepped down in the subsequent stages. Once intensity declines below the AnT, the blood lactate concentration falls until the appearance-disappearance balance is attained at the highest point of the reverse plot between intensity vs blood lactate concentration. According to the RLT proponent, this point denotes the AnT intensity. Apart from the interesting application, this original study was underpowered by the reduced sample evaluated.

Regarding the comparison between the incremental protocol and the RLT, Wahl et al., [16] showed (among other results) high accuracy between these applications to determine the MLSS. However, these authors proposed a modified RLT protocol, and the replication of the Dotan [15] findings is still required, mainly considering a larger sample. Additionally, despite heart rate is a valuable tool to link the laboratory results to field conditions [17, 18], the effect of the heart rate for AnT identification via the RLT was not tested. Due to its non-invasive nature, this analysis represents a valuable tool for athletes and non-athletes. During an incremental protocol blood lactate concentration commonly present a curvilinear kinetic [19] while heart rate increases linearly or often presents an exponential decrement [20]. However, during the reverse segment of the RLT, the heart rate presents a smoothed fashion [15] that theoretically enables the identification of AnT by the heart rate. Similar procedure was already conducted for healthy [21] and disabled [22] individuals in the lactate minimum test [23], which in turn shares some similarity within the RLT protocol. In order to increase the RLT applicability, we investigated the AnT identification during this protocol using the heart rate.

Therefore, the first aim of this study was to compare in cycling exercise the AnT determined by distinct mathematical models on traditional incremental protocol with those from the RLT. Secondarily, we aimed to test an alternative and non-invasive proposal for the AnT determination in terms of heart rate during the RLT. Moreover, both aims were tested considering the effect of the previous experience in cycling exercise.

## Materials and methods

Subjects were asked to keep the same individual hydration/food habits and avoid hard physical activity, alcohol and caffeine ingestion at least 96 hours prior the tests. Sample size estimation was performed using the G-Power software [24], considering 1-β = 0.90 and α = 0.05. According to it, ten subjects per group were enough for the aims of this study. Therefore, twenty-four active, non-athletes, and non-smokers men were divided in two groups (12 per group). Group 1 consisted of individuals (age = 24±2 years, body mass = 78.1±11.9 kg, height = 178±1 cm, body fat = 8.1±5.3%) that have a minimum of two years’ experience (3 times a week for at least 60 minutes) in cycling exercise, including mountain bike and speed subcategories. On the other hand, in Group 2 were included individuals (age = 23±3 years, body mass = 67.9±7.8 kg, height = 173±1 cm, body fat = 7.9±4.3%) that perform (3 times a week for at least 60 minutes) distinct activities (e.g. resisted training, soccer, basketball, etc.), but did not have any experience in cycling exercise. The level of physical activity was analyzed by the International Physical Activity Questionnaire (IPAQ) (Group 1 = 4985±3196 MET-min/wk; Group 2 = 3218±1803 MET-min/wk). Participants provided written, informed consent authorizing their participation in this study. All experiments were approved by the Faculty of Medical Sciences Ethics Committee (n°41257314.5.0000.5404) and were conducted according to the ethical international standards.

### Design

Twenty four subjects completed three visits to the laboratory, 36–48 hr apart. The first visit was conducted as a familiarization session (i.e. 30 minutes of cycling at fixed 80 rpm) and also for the anthropometric evaluation. During the second session, participants underwent an incremental exercise testing for AnT determination. Such procedure was necessary for the third evaluation session, in which the RLT protocol was carried with basis on the AnT. Regarding our first aim, we compared the AnT from a traditional incremental protocol with those from the RLT, considering the previous cycling experience effect. In light of our positive results, we tested the possibility to determine non-invasively the AnT from the RLT protocol in terms of heart rate. The previous experience effect was also considered in such perspective. The approaches from Cohen, Bland and Altman, Pearson and Fisher were used for analysis of magnitude of differences, agreement, relationship and comparison of variances [25–27].

### Procedures

All procedures were conducted in a controlled environment throughout experiment (temperature = 22°C±1°C; relative humidity = 50%±2%; luminosity = ~300lx. The protocols were carried on a Monark cycle ergometer (Monark Ergomedic 894 E, Monark, Sweden) at the same time of day. While experienced cyclists (Group 1) prefer high cadences (>85 rpm), non-experienced (Group 2) have opposite preferences (<75 rpm) [28]. However, evidences conclude the delta efficiency (i.e. indirect measurement of muscular efficiency) seems to be not affected by the cycling experience [28]; therefore, we opted for the 80 rpm, since it is an intermediary cadence between experienced and non-experienced individuals. The power was directly analyzed via a module USB 6008 (National Instruments, TX, US) connected to the cycle ergometer. Throughout testing, signals were collected at 1000 Hz frequency. Thereafter, were processed and analyzed using LabView-Signal-Express 2.0 (National Instruments, TX, US) and Matlab (Mathworks, MA, US), respectively. The power was calculated as: Power = (RF (EDT x PR))/6.12, where RF = resistance on the flywheel (kg); EDT = effective distance travelled (m), which was equal to 6.03 (i.e. circumference of the flywheel resistance track—1.625 m times the revolutions on the flywheel—3.714 resulted from one complete revolution); PR = pedal revolution; 6.12 is the constant for the conversion of kpm to watts [29].

### Aim 1 –Comparison between the incremental protocol and RLT results in experienced and non-experienced subjects

The incremental protocol started at initial power output of 25 W, with increments of 25 W at every 3 minutes. The exhaustion criteria were considered as non-maintenance of the predicted cadence, attainment of the predicted maximum heart rate ((210)—age*0,65) [30] or volitional exhaustion. Subjects were instructed to maintain the 80 rpm cadence throughout the test. At the end of each stage, capillarized blood samples (25 μl) were taken from the earlobe and deposited into microtubes (Eppendorf 1.5 ml) containing 50 μl of NaF. The blood L-lactate concentration was analyzed by the electrochemical method using a lactimeter YSI-2300-STAT-Plus (Yellow Springs, OH, USA).

Three mathematical models were used to calculate the AnT and blood lactate/heart rate at the AnT. Regarding the bissegmentation method (i.e. two-segment linear fit) [8, 9], blood lactate concentration was plotted against power and the lactate breakpoint was visually identified by three independent and experienced researchers; subsequently, the AnT (AnT_Inc-Biss_) was determined by the intersection of the two linear fit. The blood lactate concentration at anaerobic threshold intensity ([Lac]_AnT-Inc-Biss_) was calculated by linear interpolation. The mean heart rate of each stage was plotted against power and the heart rate at anaerobic threshold intensity (HR_AnT-Inc-Biss_) was calculated by linear interpolation.

For obtainment of AnT and heart rate at AnT by the onset of blood lactate concentration (OBLA) [10], the fixed blood lactate concentration at 4 (AnT_Inc-OBLA4_ and HR_AnT-Inc-OBLA4_) and 3 mmol.L^-1^ (AnT_Inc-OBLA3_ and HR_AnT-Inc-OBLA3_) were calculated by an exponential interpolation on the blood lactate concentration and power curve. The blood lactate concentration at these intensities was not calculated in this method since it uses fixed blood lactate concentration. The method defined as the maximal distance between two end points of the blood lactate concentration vs power curve (Dmax) [11] was also conducted. Therefore, the AnT was calculated as the maximal perpendicular distance between a linear regression (considering the two end points) and an exponential (AnT_Inc-DmaxExp_) or 2° order polynomial (AnT_Inc-DmaxPoli_) adjustments. The blood lactate concentration at these intensities were calculated by exponential ([Lac]_Inc-DmaxExp_) or polynomial ([Lac]_Inc-DmaxPoli_) interpolation.

The time to exhaustion (T_ex_) was considered as the total time of exercise performed during the incremental protocol. Heart rate was monitored using a validated monitor [31] (Polar, RS800, RJ, BR; accuracy ± 1%). As previously described for HR_AnT-Inc-Biss_ analysis, the mean heart rate of each stage was plotted against power, and the HR_AnT-Inc-OBLA4_, HR_AnT-Incremental-OBLA3_, HR_Inc-DmaxExp_ and HR_Inc-DmaxPoli_ were calculated by linear interpolation. In addition, the maximal heart rate was obtained as the highest heart rate registered during the test (HR_max-calc_) and by predicted equation (HR_max-predic)_ (HR_max-predic_ = 210—age x 0.65) [30]. The percent of maximal heart rate at the thresholds was also calculated considering the HRmax (%HR_max-calc_) and by the predicted maximum heart rate (%HR_max-predic_). Pulse oximetry (SpO_2_) was also monitored during the test (OXIFAST, Takaoka, SP, BR).

**Fig 1:**
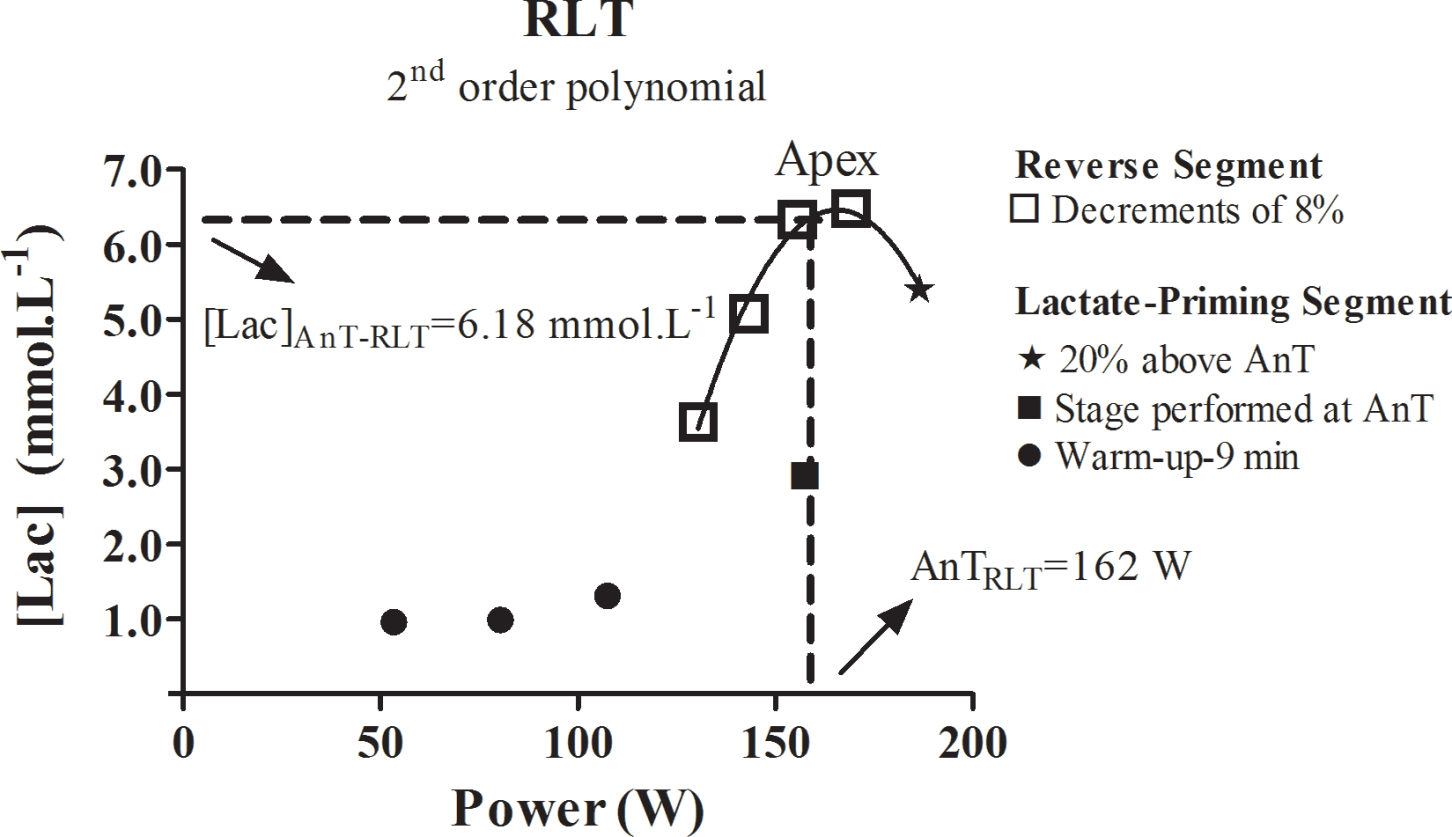
Curve of the reverse lactate threshold test considering the blood lactate concentration plotted against power from experienced individual (subject 1 from experienced group). The second order polynomial adjustment was considered for the anaerobic threshold determination (AnT_RLT_) and blood lactate concentration at anaerobic threshold ([Lac]_AnT-RLT_).

The RLT protocol was applied according to the original study [15]. The only difference consisted on the stages duration. Dotan [15] opted by the 4-min duration to avoid conflicting considerations that shorter on longer durations may result. Since previous reports demonstrated no difference in terms of AnT determination in graded exercise tests [3] within stages of 3-min or 4-min, we opted for the former to avoid lengthen evaluations. The RLT was conducted with basis on the AnT_Inc_. Capillarized blood samples were collected at the end of each stage and blood lactate concentration was analyzed as earlier described. Heart rate and pulse oximetry were also monitored. The RLT was applied in 27 minutes of cycling exercise in two phases: a) Lactate-priming segment; and b) Reverse Segment (Fig 1).

During the lactate-priming segment three graded stages below the AnT_Inc_ were conducted as a controlled warm-up. For instance, whether a subject achieved an AnT_Inc_ of 150 W, we considered the intensities of 75, 100, 125 W during the first part of the RLT lactate-priming segment (totalizing 9 minutes of warm-up). Subsequently, individuals performed three minutes in its AnT intensity and right after another stage at 20% above the AnT_Inc_. In summary, the lactate-priming segment consisted in 15 minutes of cycling.

The reverse segment was conducted following the lactate-priming segment. In this phase, intensity is retrograde. Decrements of 10 W (8%) were applied at each stage, totaling 12 minutes of cycling exercise. Considering the blood lactate concentration is elevated at the last part of the lactate-priming segment (i.e 20% above AnT_Inc_), it is expected that this metabolite decays once exercise intensity was below AnT_Inc_ (i.e. during the last two stages of the reverse segment). The reverse plot between power and blood lactate concentration was traced using a second order polynomial function. According to the RLT proponent, the AnT from the RLT protocol (AnT_RLT_) corresponds to the apex of the blood lactate concentration vs power curve. The second order polynomial equation (y = ax^2^- bx + c) was considered, and the AnT_RLT_ was calculated as y = b / (2*a), and the blood lactate concentration at AnT_RLT_ ([Lac]_AnT-RLT_) as: y = ((a* (AnT_RLT_ * AnT_RLT_))—(b * AnT_RLT_) + c). The HR_max-calc_, %HR_max-calc_, %HR_max-predic_ were also calculated. The HR_max-predic_ was the same for both RLT and incremental protocol, since considers the subject’s age [30].

### Aim 2 –Non-invasive determination of the AnT_RLT_ based on heart rate in experienced and non-experienced subjects

**Fig 2:**
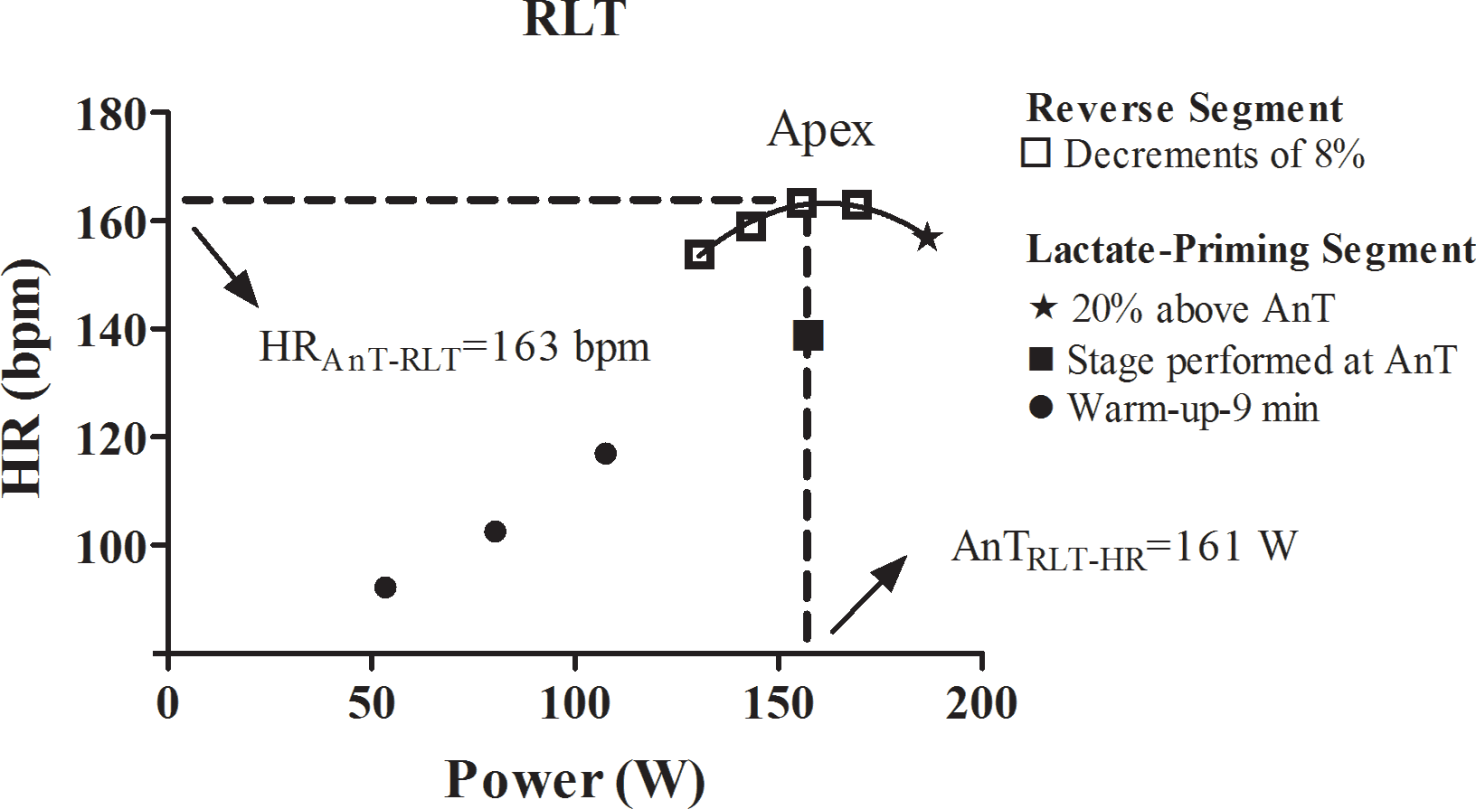
Curve of the reverse lactate threshold test considering the heart rate plotted against power from experienced individual (subject 1 from experienced group). The second order polynomial adjustment was considered for the anaerobic threshold determination (AnT_RLT-HR_) and heart rate at anaerobic threshold (HR_AnT-RLT_).

During the RLT the heart rate was monitored. The heart rate was plotted against power (Fig 2) and a second order polynomial function was adopted and the apex was considered as the AnT_RLT_ in terms of heart rate (AnT_RLT-HR_). As described in the latter section, the second order polynomial equation (y = ax^2^- bx + c) was considered, and the AnT_RLT-HR_ was calculated as y = b / (2*a), and the heart rate at AnT_RLT_ (HR_AnT-RLT_) as: y = ((a* (AnT_RLT-HR_ * AnT_RLT-HR_))—(b * AnT_RLT-HR_) + c). The success rate of the AnT_RLT_ and AnT_RLT–HR_ was determined considering the goodness of fit (R^2^) of the polynomial adjustment higher than 0.90.

### Statistical analyses

Statistical procedures were conducted using a statistical software package (STATISTICA 7.0, Statsoft, OK, USA). Mean and standard deviation (SD) were calculated for all studied variables. Levene and Shapiro-Wilk tests confirmed the homogeneity and normality of our data. Comparison among the results of the incremental protocol (i.e. AnT_Inc_, [Lac]_AnT-Inc_ and HR_AnT-Inc_, SpO2) and RLT (i.e. AnT_RLT_ and [Lac]_AnT-RLT_ and HR_AnT-RLT_, SpO2) for both experienced and non-experienced subjects was performed by the two-way ANOVA. Similar procedures were adopted for the comparison among the AnT determined by blood lactate concentration and heart rate in the RLT. In the latter case, the data from the RLT in terms of blood lactate concentration was also used in the second aim of this study. The Scheffé post-hoc was considered in all analysis of variance. The comparison of the T_ex_ between experienced and non-experienced individuals was performed by a t-test for independent samples. The same test was adopted for the comparison of the anthropometric characteristics. The agreement between variables was analyzed by the Bland-Altman procedure [25] considering α = 0.05. Pearson product moment (r) coefficient of variation (CV) [27] and effect sizes (ES) [26] were also calculated. Cohen’s categories used to evaluate the magnitude of the ES were: small if 0 ≤ |d|≤0.5; medium if 0.5 < |d| ≤ 0.8; and large if |d| > 0.8). In all cases, statistical significance was set at *P*<0.05.

## Results

### Aim 1 –Comparison between the incremental protocol and RLT results in experienced and non-experienced subjects

Statistical difference between groups regarding anthropometric characteristics was only visualized for body mass (age-*P* = 0.251; body mass-*P* = 0.017; height-*P* = 0.079; body fat-*P* = 0.982). ANOVA did not point difference regarding the mathematical models used for anaerobic threshold intensity determination on the incremental protocol and individual’s cycling experience (*P* = 0.395). Similar results were obtained for blood lactate concentration (*P* = 0.818) and heart rate (*P* = 0.469) at the anaerobic threshold intensity.

**Fig 3:**
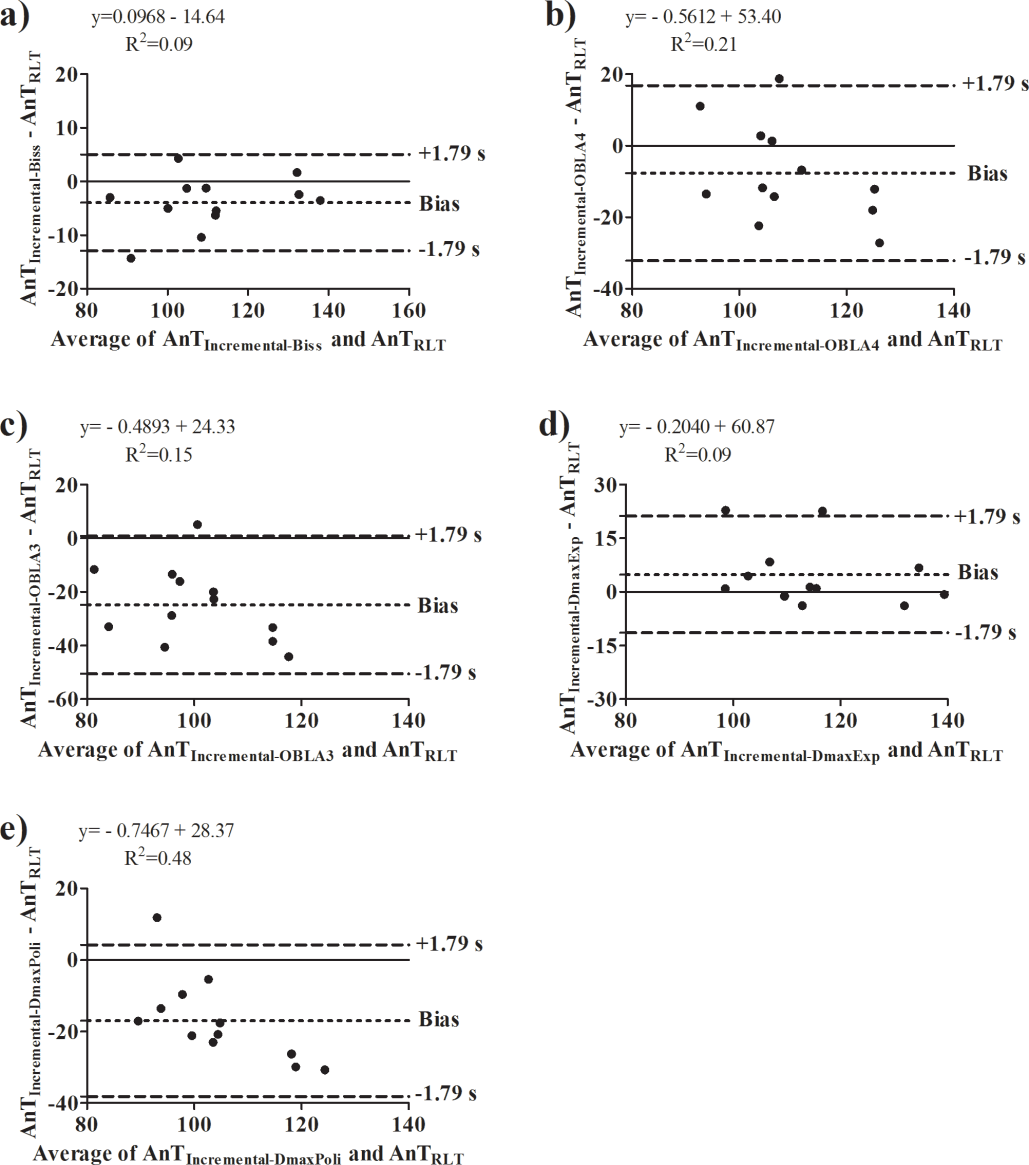
Bland-Altman analysis performed for anaerobic threshold intensity determined on the reverse lactate threshold test and incremental protocol on non-experienced individuals. **a)** Agreement between anaerobic threshold intensity determined on incremental protocol by the bissegmentation method (AnT_Inc-Biss_) and reverse lactate threshold test (AnT_RLT_); **b)** Agreement between anaerobic threshold intensity determined on incremental protocol by fixed blood lactate concentration at 4 mmol.L^-1^ (AnT_OBLA4_) and AnT_RLT_; **c)** Agreement between anaerobic threshold intensity determined on incremental protocol by fixed blood lactate concentration at 3 mmol.L^-1^ (AnT_OBLA3_) and AnT_RLT_; **d)** Agreement between anaerobic threshold intensity determined on incremental protocol by maximal deviation method using the exponential adjustment (AnT_DmaxExp_) and AnT_RLT_; **e)** Agreement between anaerobic threshold intensity determined on incremental protocol by maximal deviation method using the second order polynomial adjustment (AnT_DmaxPoli_) and AnT_RLT_.

**Fig 4:**
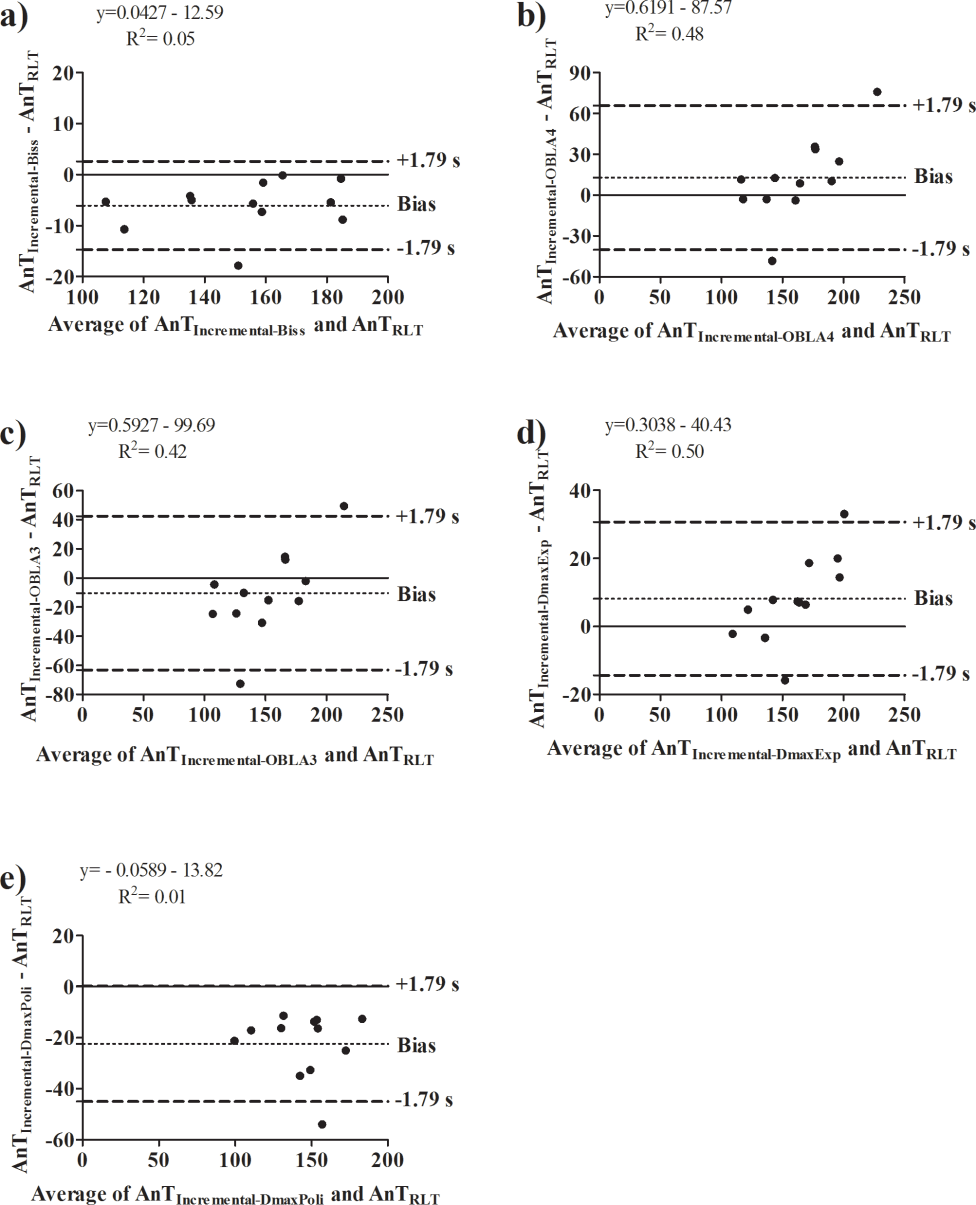
Bland-Altman analysis performed for anaerobic threshold intensity determined on the reverse lactate threshold test and incremental protocol on experienced individuals. **a)** Agreement between anaerobic threshold intensity determined on incremental protocol by the bissegmentation method (AnT_Inc-Biss_) and reverse lactate threshold test (AnT_RLT_); **b)** Agreement between anaerobic threshold intensity determined on incremental protocol by fixed blood lactate concentration at 4 mmol.L^-1^ (AnT_OBLA4_) and AnT_RLT_; **c)** Agreement between anaerobic threshold intensity determined on incremental protocol by fixed blood lactate concentration at 3 mmol.L^-1^ (AnT_OBLA3_) and AnT_RLT_; **d)** Agreement between anaerobic threshold intensity determined on incremental protocol by maximal deviation method using the exponential adjustment (AnT_DmaxExp_) and AnT_RLT_; **e)** Agreement between anaerobic threshold intensity determined on incremental protocol by maximal deviation method using the second order polynomial adjustment (AnT_DmaxPoli_) and AnT_RLT_.

In regard of the comparison between incremental protocol and RLT results (Tables 1–3), no difference was found between the anaerobic threshold intensity determined by the RLT and the incremental protocol; such result was not affected by the previous experience in cycling exercise (Table 1). However, only the comparison between AnT_RLT_ and AnT_Inc-Biss_ converge in most of statistical analysis for both non-experienced and experienced individuals. In line with this, the agreement between these intensities were the strongest when compared with the other mathematical methods (Fig 3A, Fig 4A).

**Table 1:** Comparison between the anaerobic threshold intensity from the incremental protocol analyzed by different mathematical models and the reverse lactate threshold test obtained by subjects without (non-experienced) or with (experienced) experience with cycling exercise.

	AnT_RLT_	AnT_Inc-Biss_	AnT_Inc-OBLA4_	AnT_Inc-OBLA3_	AnT_Inc-DmaxExp_	AnT_Inc-DmaxPoli_
	(W)	(W)	(W)	(W)	(W)	(W)
Non-Experienced (n = 12)						
**Mean**	112	108	105	87	117	95
**SD**	15	17	10	10	13	8
***P* (Scheffé)**	———	0.977	0.924	0.180	0.967	0.226
**ES**	———	0.24	0.60	1.86	0.34	1.42
**% Diff**	———	3.4	6.8	21.9	4.3	15.1
**r (*P*)**	———	0.95 (0.008)	0.50 (0.090)	0.46 (0.127)	0.81 (0.001)	0.67 (0.015)
**CV**	———	3.1	8.4	10.1	5.6	8.0
**Bland-Altman**	———	-3.9±5.0	-7.6±13.6	-24.7±14.35	4.8±9.1	-16.9±11.84
Experienced (n = 12)						
**Mean**	155	149	167	143	164	133
**SD**	25	26	41	39	34	23
***P* (Scheffé)**	———	0.925	0.722	0.824	0.868	0.062
**ES**	———	0.23	0.34	0.37	0.27	0.91
**% Diff**	———	3.8	7.2	7.6	5.2	14.3
**r (*P*)**	———	0.98 (0.000)	0.79 (0.002)	0.75 (0.004)	0.95 (0.000)	0.87 (0.000)
**CV**	———	2.2	12.3	12.5	5.6	6.2
**Bland-Altman**	———	-6.0±4.8	11.3±26.3	-11.9±26.4	8.1±12.5	-22.3±12.6

Note: The statistical results refers to the comparison between the anaerobic threshold intensity from the reverse lactate threshold and the anaerobic threshold intensity from the mathematical models applied in the incremental protocol. AnT_RLT_—Anaerobic threshold intensity determined during the reverse lactate threshold test in terms of blood lactate concentration. AnT_Inc-Biss_−Anaerobic threshold intensity determined during the incremental protocol by the bissegmentation method; AnT_Inc-OBLA4_ –Anaerobic threshold intensity determined during the incremental protocol by the fixed blood lactate concentration at 4 mmol.L^-1^; AnT_Inc-OBLA3_ –Anaerobic threshold intensity determined during the incremental protocol by the fixed blood lactate concentration at 3 mmol.L^-1^; AnT_Inc-DmaxExp_−Anaerobic threshold intensity determined during the incremental protocol by maximal deviation method using the exponential adjustment; AnT_Inc-DmaxPoli_−Anaerobic threshold intensity determined during the incremental protocol by maximal deviation method using the second order polynomial adjustment; *P* (Scheffé)–*P* value from the Scheffé post-hoc analysis; ES–effect size; % Diff–percent difference between means; r (*P*) Pearson product moment and *P* value from the correlation; CV–coefficient of variation; Bland-Altman—Bland Altman analysis considering 95% of agreement limits.

**Table 2:** Comparison between the blood lactate concentration at the anaerobic threshold intensity from the incremental protocol analyzed by different mathematical models and the reverse lactate threshold test obtained by subjects without (non-experienced) or with (experienced) experience with cycling exercise.

	[Lac]_AnT-RLT_	[Lac]_AnT-Inc-Biss_	[Lac]_AnT-Inc-OBLA4_	[Lac]_AnT-Inc-OBLA3_	[Lac]_AnT-Inc-DmaxExp_	[Lac]_AnT-Inc-DmaxPoli_
	(mmol.L^-1^)	(mmol.L^-1^)	(mmol.L^-1^)	(mmol.L^-1^)	(mmol.L^-1^)	(mmol.L^-1^)
Non-Experienced (n = 12)						
**Mean**	6.74	3.26	———	———	5.01	3.20
**SD**	2.63	1.29	———	———	1.03	0.86
***P* (Scheffé)**	———	0.013	———	———	0.417	0.009
**ES**	———	1.78	———	———	0.95	2.03
**% Diff**	———	51.6	———	———	25.6	52.5
**r (*P*)**	———	0.71 (0.008)	———	———	0.70 (0.010)	0.54 (0.065)
**CV**	———	27.3	———	———	24.6	32.4
**Bland-Altman**	———	-3.4±1.9	———	———	-1.7±2.0	-3.5±2.2
Experienced (n = 12)						
**Mean**	6.47	2.27	———	———	4.12	1.88
**SD**	3.84	1.08	———	———	1.46	0.85
***P* (Scheffé)**	———	0.002	———	———	0.166	0.000
**ES**	———	1.71	———	———	0.89	1.95
**% Diff**	———	64.9	———	———	36.2	70.8
**r (*P*)**	———	0.29 (0.350)	———	———	0.38 (0.212)	0.07 (0.816)
**CV**	———	59.4	———	———	47.3	77.6
**Bland-Altman**	———	-4.2±3.6	———	———	-2.3±3.5	-4.5±3.9

Note: The statistical results refers to the comparison between the anaerobic threshold intensity from the reverse lactate threshold and the anaerobic threshold intensity from the mathematical models applied in the incremental protocol. [Lac]_AnT-RLT_−Blood lactate concentration at the anaerobic threshold intensity determined during the reverse lactate threshold test; [Lac]_AnT-Inc-Biss_−Blood lactate concentration at the anaerobic threshold intensity determined during the incremental protocol by the bissegmentation method; [Lac]_AnT-Inc-OBLA4_ –Blood lactate concentration at the anaerobic threshold intensity determined during the incremental protocol by the fixed blood lactate concentration at 4 mmol.L^-1^; [Lac]_AnT-Inc-OBLA3_ –Blood lactate concentration at the anaerobic threshold intensity determined during the incremental protocol by the fixed blood lactate concentration at 3 mmol.L^-1^; [Lac]_AnT-Inc-DmaxExp_−Blood lactate concentration at the anaerobic threshold intensity determined during the incremental protocol by maximal deviation method using the exponential adjustment; [Lac]_AnT-Inc-DmaxPoli_−Blood lactate concentration at the anaerobic threshold intensity determined during the incremental protocol by maximal deviation method using the second order polynomial adjustment; *P* (Scheffé)–*P* value from the Scheffé post-hoc analysis; ES–effect size; % Diff–percent difference between means; r (*P*) Pearson product moment and *P* value from the correlation; CV–coefficient of variation; Bland-Altman—Bland Altman analysis considering 95% of agreement limits.

**Table 3:** Comparison between the heart rate at the anaerobic threshold intensity from the incremental protocol analyzed by different mathematical models and the reverse lactate threshold test obtained by subjects without (non-experienced) or with (experienced) experience with cycling exercise.

	HR_AnT-RLT_	HR_AnT-Inc-Biss_	HR_AnT-Inc-OBLA4_	HR_AnT-Inc-OBLA3_	HR_AnT-Inc-DmaxExp_	HR_AnT-Inc-DmaxPoli_
	(bpm)	(bpm)	(bpm)	(bpm)	(bpm)	(bpm)
Non-Experienced (n = 12)						
**Mean**	171	155	152	144	159	148
**SD**	13	13	15	15	14	13
***P* (Scheffé)**	———	0.000	0.001	0.000	0.010	0.000
**ES**	———	1.21	1.30	1.89	0.89	1.72
**% Diff**	———	9.4	10.9	15.8	7.2	13.5
**r (*P*)**	———	0.64 (0.022)	0.42 (0.165)	0.35 (0.255)	0.45 (0.138)	0.37 (0.226)
**CV**	———	4.8	3.5	7.4	6.2	6.6
**Bland-Altman**	———	-16.1±11.1	-18.7±15.4	-27.1±16.4	-12.3±14.5	-23.1±15.0
Experienced (n = 12)						
**Mean**	177	159	165	155	163	150
**SD**	12	10	14	13	11	8
***P* (Scheffé)**	———	0.007	0.321	0.007	0.088	0.000
**ES**	———	1.62	0.88	1.71	1.17	2.63
**% Diff**	———	10.4	6.6	12.3	7.8	15.2
**r (*P*)**	———	0.44 (0.146)	0.09 (0.760)	0.01 (0.959)	0.14 (0.659)	0.03 (0.907)
**CV**	———	5.1	4.1	7.8	6.4	6.3
**Bland-Altman**	———	-18.6±12.5	-11.7±19.9	-21.9±18.3	-13.8±15.5	-27.0±14.5

Note: The statistical results refers to the comparison between the anaerobic threshold intensity from the reverse lactate threshold and the anaerobic threshold intensity from the mathematical models applied in the incremental protocol. HR_AnT-RLT_−Hear rate at the anaerobic threshold intensity determined during the reverse lactate threshold test; HR_AnT-Inc-Biss_−Heart rate at the anaerobic threshold intensity determined during the incremental protocol by the bissegmentation method; HR_AnT-Inc-OBLA4_ –Hear rate at the anaerobic threshold intensity determined during the incremental protocol by the fixed blood lactate concentration at 4 mmol.L^-1^; HR_AnT-Inc-OBLA3_ –Hear rate at the anaerobic threshold intensity determined during the incremental protocol by the fixed blood lactate concentration at 3 mmol.L^-1^; HR_AnT-Inc-DmaxExp_−Hear rate at the anaerobic threshold intensity determined during the incremental protocol by maximal deviation method using the exponential adjustment; HR_AnT-Inc-DmaxPoli_−Hear rate at the anaerobic threshold intensity determined during the incremental protocol by maximal deviation method using the second order polynomial adjustment; *P* (Scheffé)–*P* value from the Scheffé post-hoc analysis; ES–effect size; % Diff–percent difference between means; r (*P*) Pearson product moment and *P* value from the correlation; CV–coefficient of variation; Bland-Altman—Bland Altman analysis considering 95% of agreement limits.

Apart from the non-difference (for both groups) and significant relationship (only for non-experienced subjects) between [Lac]_AnT-RLT_ and [Lac]_AnT-Inc-DmaxExp_, distinct results were found between protocols in terms of the blood lactate concentration at the AnT (Table 2). Excepted of the significant relationship between HR_AnT-RLT_ and HR_AnT-Inc-Biss_, different results were found between protocols for heart rate at the AnT intensity (Table 3). In addition, the Table 4 shows the results of HR_max-calc_, HR_max-predic_, %HR_max-calc_, %HR_max-predic_. The T_ex_ was different between non-experienced (18min 13s ± 1min 46s) and experienced (22min 54s ± 2min 48s) individuals (*P* = 0.000). Pulse oximetry was not different during both incremental (non-experienced– 96.4±1.0; experienced– 96.1±1.2%; *P =* 0.916) and RLT (non-experienced– 96.0±1.0; experienced– 95.4±1.2%; *P =* 0.695); additionally, ANOVA also did not show interaction regarding the factorials variables (i.e. test and experience) (*P* = 0.729).

**Table 4:** Maximal heart rate predicted (HR_max-predic_) and registered (HR_max-calc_) during the reverse lactate threshold test and incremental protocol, as well as the percent of maximal heart rate at the anaerobic threshold intensities considering the registered (%HR_max-calc_) and predicted (%HR_max-predic_) maximal heart rate.

			Non-Experienced (n = 12)	Experienced (n = 12)
**RLT**		HR_max-calc_ (bpm)	177±14	183±13
		HR_max-predic_ (bpm)	197±3	195±2
	**AnT**_**RLT**_	%HR_max-calc_	96±1	96±1
		%HR_max-predic_	91±7	94±6
**Incremental**		HR_max-calc_ (bpm)	191±16	195±7
		HR_max-predic_ (bpm)	197±3	195±2
	**AnT**_**Inc-Biss**_	%HR_max-calc_	81±6	81±5
		%HR_max-predic_	80±7	81±5
	**AnT**_**Inc-OBLA4**_	%HR_max-calc_	79±5	85±8
		%HR_max-predic_	78±8	85±7
	**AnT**_**Inc-OBLA3**_	%HR_max-calc_	76±6	79±7
		%HR_max-predic_	74±8	80±7
	**AnT**_**Inc-DmaxExp**_	%HR_max-calc_	83±5	83±5
		%HR_max-predic_	81±7	84±6
	**AnT**_**Inc-DmaxPoli**_	%HR_max-calc_	77±4	77±5
		%HR_max-predic_	76±7	77±4

AnT_RLT_—Anaerobic threshold intensity determined during the reverse lactate threshold test in terms of blood lactate concentration. AnT_Inc-Biss_−Anaerobic threshold intensity determined during the incremental protocol by the bissegmentation method; AnT_Inc-OBLA4_ –Anaerobic threshold intensity determined during the incremental protocol by the fixed blood lactate concentration at 4 mmol.L^-1^; AnT_Inc-OBLA3_ –Anaerobic threshold intensity determined during the incremental protocol by the fixed blood lactate concentration at 3 mmol.L^-1^; AnT_Inc-DmaxExp_−Anaerobic threshold intensity determined during the incremental protocol by maximal deviation method using the exponential adjustment; AnT_Inc-DmaxPoli_−Anaerobic threshold intensity determined during the incremental protocol by maximal deviation method using the second order polynomial adjustment

### Aim 2 –Non-invasive determination of the AnT_RLT_ based on heart rate in experienced and non-experienced subjects

**Fig 5:**
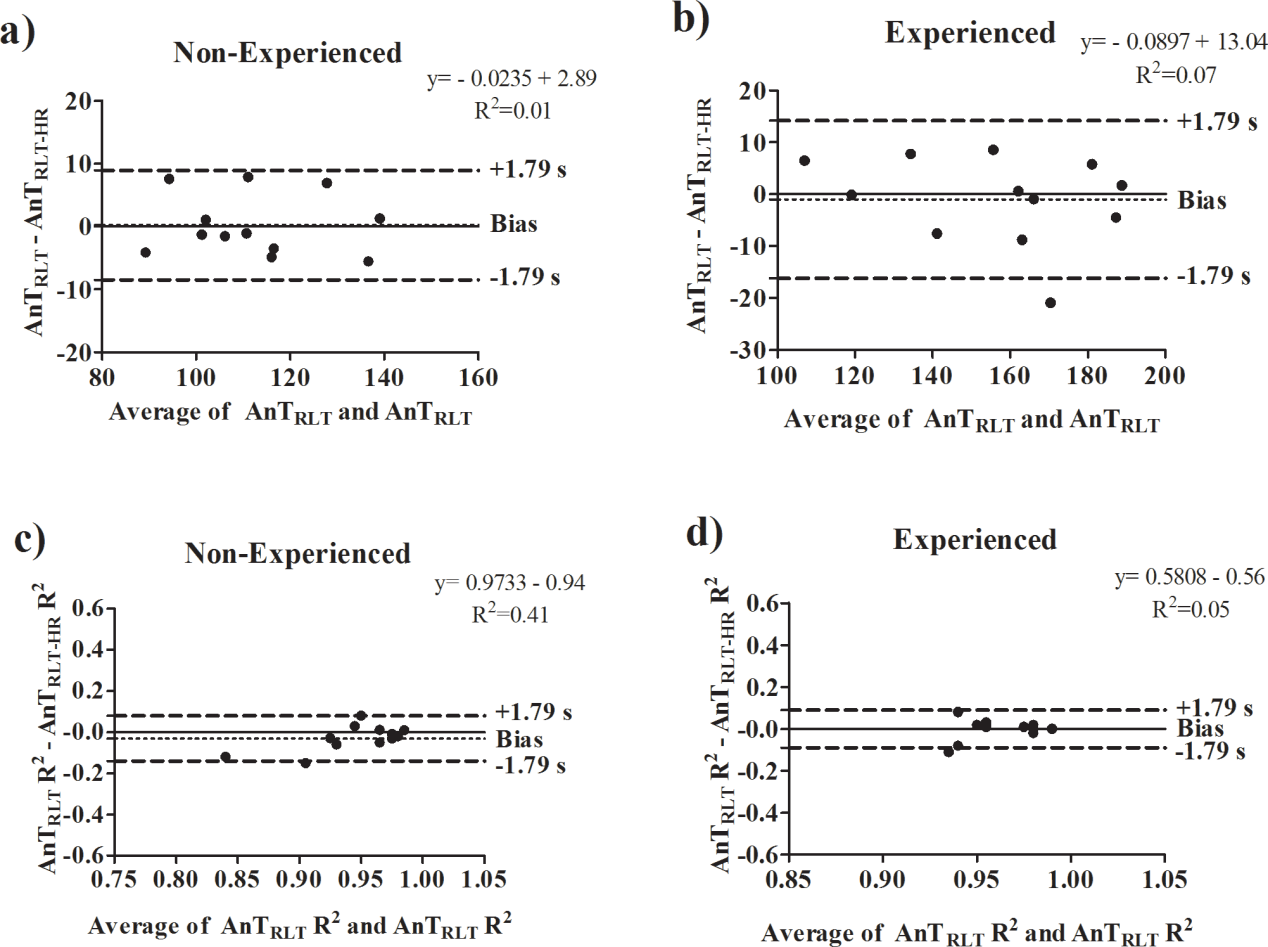
**a)** Agreement performed by Bland-Altman analysis for anaerobic threshold intensity determined on the reverse lactate threshold test by blood lactate concentration (AnT_RLT_) and heart rate (AnT_RLT-HR_) for non-experienced individuals; **b)** Agreement performed by Bland-Altman analysis AnT_RLT_ and AnT_RLT-HR_ for experienced individuals; **c)** Agreement performed by Bland-Altman analysis for coefficient of determination on the reverse lactate threshold test by blood lactate concentration (AnT_RLT_ R^2^) and heart rate (AnT_RLT-HR_ R^2^) for non-experienced individuals; **d)** Agreement performed by Bland-Altman analysis AnT_RLT_ R^2^ and AnT_RLT-HR_ R^2^ for experienced individuals.

The Table 5 shows the AnT determined during the RLT by means of heart rate for both groups. All statistical procedures converged to the similarity between the results, with the exception of the non-significant relationship (for both groups). The agreement among variables are shown on Fig 5. High R^2^ were obtained on the polynomial adjustment adopted for both groups in terms of AnT_RLT_ (Non-Experienced—range = 0.78–0.99; Experienced—range = 0.90–0.99) and AnT_RLT-HR_ (Non-Experienced—range = 0.90–0.99; Experienced—range = 0.90–0.99). In line with this, high success rate were obtained for the two determinations in both groups.

**Table 5:** Comparison between the results from the reverse lactate threshold test analyzed in terms of blood lactate concentration and heart rate obtained by subjects without (non-experienced) or with (experienced) experience with cycling exercise.

	AnT_RLT_	AnT_RLT-HR_	AnT_RLT_	AnT_RLT-HR_	AnT_RLT_	AnT_RLT-HR_
	(W)	(W)	R^2^	R^2^	Success rate (%)	Success rate (%)
Non-Experienced (n = 12)					83.33 (n = 10)	100 (n = 12)
**Mean**	112	112	0.93	0.96		
**SD**	15	16	0.07	0.03		
***P* (Scheffé)**	0.999		0.331			
**ES**	0.02		0.69			
**% Diff**	0.3		3.9			
**r (*P*)**	0.95 (0.000)		0.29 (0.354)			
**CV**	3.0		5.4			
**Bland-Altman**	0.2±4.8		-0.0±0.0			
Experienced (n = 12)					91.66 (n = 11)	100 (n = 12)
**Mean**	155	156	0.96	0.97		
**SD**	25	27	0.04	0.03		
***P* (Scheffé)**	0.999		0.998			
**ES**	0.04		0.10			
**% Diff**	0.6		0.3			
**r (*P*)**	0.95 (0.000)		0.14 (0.656)			
**CV**	3.8		3.8			
**Bland-Altman**	1.0±8.4		0.0±0.0			

AnT_RLT_—Anaerobic threshold intensity determined during the reverse lactate threshold test in terms of blood lactate concentration; AnT_RLT-HR_—Anaerobic threshold intensity determined during the reverse lactate threshold test in terms of heart rate; AnT_RLT_ R^2^—goodness of fit of the polynomial adjustment between power and blood lactate concentration (reverse segment); AnT_RLT-HR_ R^2^—goodness of fit of the polynomial adjustment between power and heart rate (reverse segment); Success rate—goodness of fit of the polynomial adjustment higher than 0.90. *P* (Scheffé)–*P* value from the Scheffé post-hoc analysis; ES–effect size; % Diff–percent difference between means; r (*P*) Pearson product moment and *P* value from the correlation; CV–coefficient of variation; Bland-Altman—Bland Altman analysis considering 99% of agreement limits.

## Discussion

The main findings of the present investigation were the AnT determined on the RLT was consistent with those obtained on the incremental protocol calculate by the bissegmentation mathematical model. In addition, our results showed that previous cycling experience did not influence on such consistency. However, due to the different methodologies, additional parameters like blood lactate concentration and heart rate at the anaerobic threshold intensity are not similar. Additionally, for 83% of the evaluated non-experienced individuals the difference between the AnT determined on RLT by blood lactate concentration and heart rate was 6%. Similar difference was also obtained for 91% of the evaluated experienced individuals. Therefore, our results suggest that AnT determination via heart rate on RLT may be considered as a non-invasive alternative. However, future studies are necessary to investigate which factors can improve the agreement between these measures.

### Aim 1 –Comparison between the incremental protocol and RLT results in experienced and non-experienced subjects

Regarding our first aim, we found no difference between the AnT determined during the RLT and incremental protocol. On the other hand, differences in terms of blood lactate concentration and heart rate at AnT intensity were found. Despite these distinct results, the previous experience in cycling exercise did not affect the results provided by both protocols. Overall, both protocols seem to identify the same phenomenon (i.e. AnT) regardless of previous experience in cycling, but the physiological condition that such identification occurs depends on the characteristic of each protocol.

The main criticism suggested by Dotan [15] regarding the incremental exercise is related to the ambiguity of where the highest blood lactate concentration is identified. Some studies converge with this approach [13, 14]. Although the RLT seems to deal with such problem, its proponent also highlights other methodological concerns [15]. For instance, the need for “on-the-fly” blood sampling is suggested as a limited factor for sports where this is not possible (e.g. swimming); this limitation can be extended to incremental protocols applied to these exercises. In this sense, long and repetitive exercise breaks can lead to an AnT overestimation, since the glycolytic rate resulted from the previous incremental stage is decreased while oxygen uptake remains elevated, improving the blood lactate concentration clearance [32]. However, such limitation was not present in our study. Moreover, the duration of stages were standardized for the RLT and incremental protocol. We believe that these factors along with the RLT application based on the incremental protocol were crucial for the similarity in terms of AnT.

Indeed, the AnT may be identified during the incremental protocol when an abrupt elevation of blood lactate concentration is visualized. In this sense, the balance of blood lactate concentration between lactate producing and absorbing compartments is gradually lost. Conversely, the RLT test is conducted under an inverse concept, that is, the organism is already facing the loss of blood lactate concentration balance between compartments (i.e. last part of lactate-priming segment). When intensities are stepped down in the reverse segment, the balance between blood lactate concentration production-removal is gradually recovered. On the other hand, because at least two stages were performed above the predicted AnT (i.e. last stage from the lactate-priming segment and first stage of the reverse segment), the blood lactate concentration removal will be greater than its production only when the stages below the predicted AnT were performed. Such characteristic is responsible by the high blood lactate concentration and heart rate found in our study at the apex of the second order polynomial adjustment, that is, at the AnT_RLT_ (Tables 2–3).

According to Dotan [15], further experimentation is required before final validation of the RLT. Thus, we contributed with such perspective by demonstrating that the previous cycling experience did not affect the AnT_RLT_ identification. It is consolidated that muscular efficiency as well as aerobic and anaerobic power/capacity between individuals with or without cycling experience is quite distinct [33, 34]. Despite the encouraging reports, methodological concerns related to fitness level must be investigated. For instance, we considered the upper limit of 20% increment proposed in the original RLT during the last stage of the lactate-priming segment. However, since well-trained individuals present the AnT at high percentage of its maximal oxygen consumption (VO_2max_) [35], it is possible that the 20% increment surpass its maximal oxygen consumption intensity, leading to premature exhaustion.

Regarding the reverse segment, the individual’s physiological/metabolic condition must be also accounted. The improvement of blood lactate clearance is considered as positive physiological adaptation from endurance training [36], which can be extended to well-trained individuals. Therefore, the upper limit of 8% decrement originally proposed for the reverse segment may lead to a faster blood lactate concentration clearance in these individuals, overestimating the real AnT. Regarding the stage duration, the slight modification used in this study (i.e. 3-min instead of the original 4-min) did not implicate on the AnT_RLT_ determination, since high coefficient of determinations and high success rate were obtained. Other methodological concerns were considered in the study of Wahl et al., [16], in which a modified RLT protocol was proposed to improve the test’s precision, also enabling the VO_2max_ determination during the lactate priming-segment. Within this new approach the RLT was more accurate to determine the MLSS than other threshold concepts, such as OBLA4 [10] or the Dmax [11]. Overall, further studies should consider the methodological concerns highlighted along with the results of Wahl et al., [16], mainly whether the RLT application was conducted to highly trained individuals.

Lastly, another RLT limitation is the necessity of the previous AnT determination for intensity prescription during the two phases. However, futures studies are encouraged to investigate whether indirect methods or even the reported mean velocity during recent championships/training sessions to estimate the intensities of the RLT. The marked differences in terms of methodology and application implies in the use of the RLT and incremental protocol inside the sport and clinical contexts. For instance, evidences have been suggested that blood lactate concentration and heart rate at the AnT are valid indicators for monitoring longitudinal training effects for different populations [17, 37]. In this sense, because during the RLT application high values of [Lac]_AnT-RLT_ and HR_AnT-RLT_ are obtained, it is possible that this protocol is disadvantaged for utilization of these results for monitoring longitudinal training effects. This detriment is not transposed to the incremental protocol, since the [Lac]_AnT-Inc_ and HR_AnT-Inc_ are consolidated indicators for training control [17, 37]. Moreover, RLT does not requires exhaustion, the HR_max-calc_ and %HR_max-calc_ values cannot be used in the same way as for the incremental protocol (Table 4).

It is valid to state that the comparison among mathematical models for determining the anaerobic threshold intensity on incremental protocol is beyond the main aim of this study. Such results were provided to strength the comparison between results from the incremental protocol with the reverse lactate threshold test. In line with this, the results on Table 1 showed that only the AnT_Inc-Biss_ and the AnT_RLT_ converged in all statistical procedures adopted. In terms of Pearson product moment, both non-experienced and experienced were heterogeneous regarding AnT (non-experienced 8–15% and experienced 17–27% if considered all mathematical models), which could have improved the correlation coefficients.

In regard of the agreement, the bias from Bland-Altman analysis revealed that in some non-experienced subjects the AnT_RLT_ overestimated the AnT_Inc-Biss_ in ~9W. This result was confirmed in subjects 3 and 10, in which the AnT_RLT_ was 14 and 10 W (respectively) higher than the AnT_Inc-Biss_. However, without these subjects the bias is reduced to ~5W. Overall, for 83% of non-experienced subjects this overestimation only represents 4%. This perspective is extended for experienced subjects. In this case the bias is ~8W, which was confirmed for subjects 7, 10 and 12 (i.e. AnT_RLT_ overestimated the AnT_Inc-Biss_ in 10, 8 and 17 W, respectively). However, without these subjects the bias is reduced to ~4W, which represents an overestimation of only 2% for 75% of experienced subjects.

Although fixed blood lactate concentration enables the AnT determination without the exhaustion attainment in incremental protocols [10], some studies have demonstrated that depending on the mathematical model adopted, the AnT determination can be influenced [38, 39]. Therefore, despite the excessive blood sampling may be not suitable for practical contexts, the exhaustion attainment is interesting for the robust analysis of the AnT that is being measured. Moreover, the reliable acquisition of additional parameters (e.g. maximal oxygen consumption, velocity at maximal oxygen consumption or time to exhaustion) requires that individuals reach exhaustion. However, the exhaustion attainment necessity may be not suitable for untrained individuals. In this sense, the non-exhaustive nature of the RLT is an interesting possibility for the AnT identification in peculiar cases, such as untrained or inexperienced subjects. Therefore, although no differences between AnT_Inc_ and AnT_RLT_ were found, we suggest that future studies should consider its applications according to the population that will be evaluated.

### Aim 2 –Non-invasive determination of the AnT_RLT_ based on heart rate in experienced and non-experienced subjects

Heart rate has been considered for monitoring training since 1980s [17]. Sooner after the expansion of heart devices, Conconi et al., [40] proposed the AnT estimation based on this physiological parameter. Nowadays the individualized exercise prescription as well as load controlling based on heart rate are commonly used in practice [17, 18, 41, 42], since factors as easy applicability and reduced financial cost improve the heart rate devices utilization. Additionally, in a recent meta-analysis Bellenger et al., [41] concluded the autonomic heart rate regulation may be considered a potential indicator for overreaching status.

Since Dotan [15] demonstrated the heart rate presented a smoothed fashion during the reverse segment, it also opened for discussion if the AnT could be determined based on this physiological parameter. In accordance, our results showed the AnT is determined during RLT based on of heart rate. Moreover, this determination was not affected by the previous experience in the cycling exercise. These results improve the scientific knowledge around the RLT application, since the heart rate is a cheap and easy non-invasive tool.

The AnT determination via heart rate during the RLT was possible for all subjects, as exposed in Table 5. In fact, the success rate of AnT determination via blood lactate concentration for two non-experienced (subject 8 –R^2^ = 0.83; subject 9 –R^2^ = 0.78) and one experienced (subject 11 –R^2^ = 0.89) was slightly lower. On the other, the success rate for the AnT via heart rate was 100% for both non-experienced and experienced subjects. In fact, while the blood lactate concentration tends to decrease significantly once the equilibrium between production-removal is gradually recovery, the cardiovascular system tends to readjust after the stressful stage (i.e. 20% above the AnT) in a smoothed fashion [15](Fig 2). However, such differences did not affect the second order polynomial adjustment, since high R^2^ were found for all subjects in the AnT determination via blood lactate concentration and heart rate.

Apart from the non-difference, low effect size and significant correlation, the comparison between AnT determined from both blood lactate concentration and heart rate resulted in a bias of ~8 and ~15W in the Bland-Altman analysis for non-experienced and experienced subjects, respectively. As discussed in the previous section, such bias can be related with the results of few subjects. For instance, the difference of AnT_RLT_ and AnT_RLT-HR_ for subjects 6 and 7 (i.e. Group 1) was ~7 and ~8W, respectively. Without this difference, the bias is reduced to ~6W which represents a difference of AnT_RLT_ and AnT_RLT-HR_ of 6% for 83% of evaluated non-experienced subjects.

**Fig 6:**
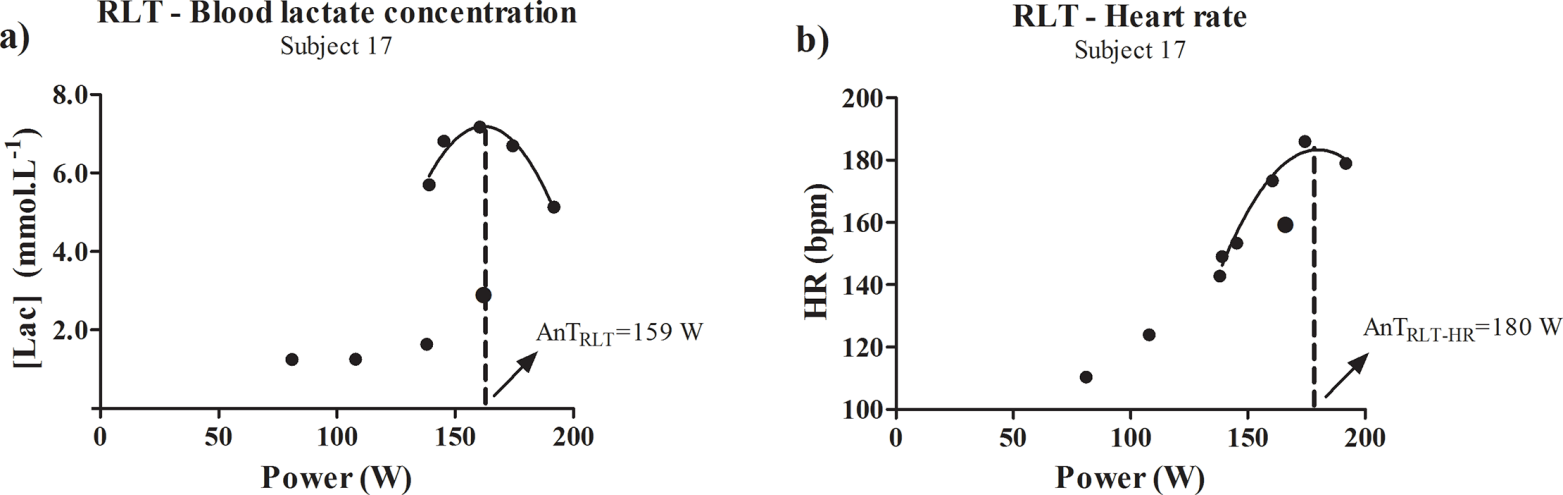
**a)** Curve of the reverse lactate threshold test from subject 5 (Experienced group) considering the blood lactate concentration plotted against power. The second order polynomial adjustment was considered for the anaerobic threshold determination (AnT_RLT_); **b)** Curve of the reverse lactate threshold test from subject 5 (Experienced group) considering the heart rate plotted against power. The second order polynomial adjustment was considered for the anaerobic threshold determination (AnT_RLT-HR_).

Regarding the experienced individuals, the bias was ~15W. For this group, the heart rate behavior during the RLT reverse segment for one experienced subject was markedly different from the others (Fig 6). It is clear to notice the high decrement of heart rate mainly in the two last stages of the test. Such behavior can be related with a low efficiency in terms of readjustment of autonomic nervous system along with the sympathetic-parasympathetic regulation on the cardiovascular system [41]. Within this result, the zero tangent in x-axis of the polynomial adjustment was right shifted, overestimating the AnT via heart rate in 13% (180 W) when compared to the blood lactate concentration determination (159 W). This overestimation is also extended when considered the AnT_Inc-Biss_ of this subject (158W). Without this subject, the bias of AnT_RLT_ and AnT_RLT-HR_ for the experienced subjects is reduced for ~10W, which means that for 91% of these individuals the difference may be 6%.

Overall, we do not believe that differences invalidate the AnT determination via heart rate during the RLT. Every proposed test to determine the AnT has a bias. The major question in this issue is related within the magnitude of the error. It is consolidated that heart rate is a valuable maker for monitoring training effects or even determine physiological phenomenon such as the AnT [17, 18, 41]. Our results corroborate with this perspective. However, every measurement must be carefully analyzed in order to identify factors that are culminating for discrepancies between methods, as demonstrated on Fig 6. Therefore, the results presented in this study strengthen the determination of AnT in the RLT via heart rate, which can a valid and useful tool.

## Conclusions

In summary, the AnT determined from the RLT is consistent with those determined from the incremental protocol by means of the bissegmentation model. However, caution is required regarding the comparison of other physiological results (blood lactate concentration and heart rate at AnT), since protocols are different considering methodology, resulting in distinct results. Additionally, the previous experience in cycling exercise did not affect the AnT from such application, highlighting the robustness of this RLT for AnT determination. Moreover, we demonstrated the AnT determination using heart rate is a valid non-invasive alternative, and it’s also not a function of the previous experience in cycling exercise. However, for 83% and 91% of the evaluated non-experienced and experienced individuals (respectively), the difference between the AnT determined on RLT by blood lactate concentration and heart rate was 6%. Therefore, future studies are necessary to investigate which factors can improve the agreement between these measures.

## Supporting information

S1 File Datasheet with all data used in the manuscript. Folder “IncrementalTestBiss” contains the data regarding the bissegmentation method. Moreover, this folder contains general information regarding the incremental protocol, such as the calculated and predicted maximal heart rate (HRmax-Incremental-calculated and HRmax-Incremental-predicted), time to exhaustion (Tex) and pulse oximetry (SpO_2_). Folder “IncrementalTestOBLA” contains the data regarding the fixed blood lactate concentration method considering 3 and 4 mmol.L^-1^. Folder “IncrementalTestDMAX” contains the data regarding the maximal perpendicular distance method considering exponential and polynomial second order adjustments. Folder “ReverseLactateThresholdTest”contains the data regarding the Reverse Lactate Threshold Test.
(XLSX)
